# Supplementary material for: Flower-like Thiourea–Formaldehyde Resin Microspheres for the Adsorption of Silver Ions
Source: Polymers (Basel). 2023 May 23;15(11):2423. doi: 10.3390/polym15112423 (PMC10255821; doi:10.3390/polym15112423)
Supplement: Supplementary file 1 [file polymers-15-02423-s001.zip › polymers-2393742-supplementary.pdf]

polymers

MDPI

Article

Flower-Like Thiourea–Formaldehyde Resin Microspheres for the Adsorption of Silver Ions

Yuhan Li [1,†], Xiaoli Wang [2,†], Jing Xia [3], Guangwei Zhou [1], Xiaomu Wang [1], Dingxuan Wang [1], Junying Zhang [1], Jue Cheng [1,*] and Feng Gao [1,*]

[1] Key Laboratory of Carbon Fiber and Functional Polymers, Ministry of Education, Beijing University of Chemical Technology, Beijing 100029, China; chn_liyh@163.com (Y.L.); zgwbuct@163.com (G.Z.); w343002449@163.com (X.W.); wangdx_2022@163.com (D.W.); zhangjy@mail.buct.edu.cn (J.Z.)
[2] Aerospace Research Institute of Materials & Processing Technology, Beijing 100076, China; wangxiaoli@arimt.com
[3] Ansteel Beijing Research Institute Co., Ltd., Beijing 102200, China; xiaj0001@126.com
* Correspondence: chengjue@mail.buct.edu.cn (J.C.); gaofeng@mail.buct.edu.cn (F.G.); Tel./Fax: +86-10-6442-5439 (J.C. & F.G.)
† These authors contributed equally to this work.

Citation: Li, Y.; Wang, X.; Xia, J.; Zhou, G.; Wang, X.; Wang, D.; Zhang, J.; Cheng, J.; Gao, F. Flower-Like Thiourea–Formaldehyde Resin Microspheres for the Adsorption of Silver Ions. *Polymers* **2023**, *15*, 2423. https://doi.org/10.3390/polym15112423

Academic Editors: Xin Zhou, Wei Zhang, Peng Zhang, Bin Ji and Shujuan Meng

Received: 26 April 2023
Revised: 14 May 2023
Accepted: 17 May 2023
Published: 23 May 2023

Copyright: © 2023 by the authors. Licensee MDPI, Basel, Switzerland. This article is an open access article distributed under the terms and conditions of the Creative Commons Attribution (CC BY) license (https://creativecommons.org/licenses/by/4.0/).

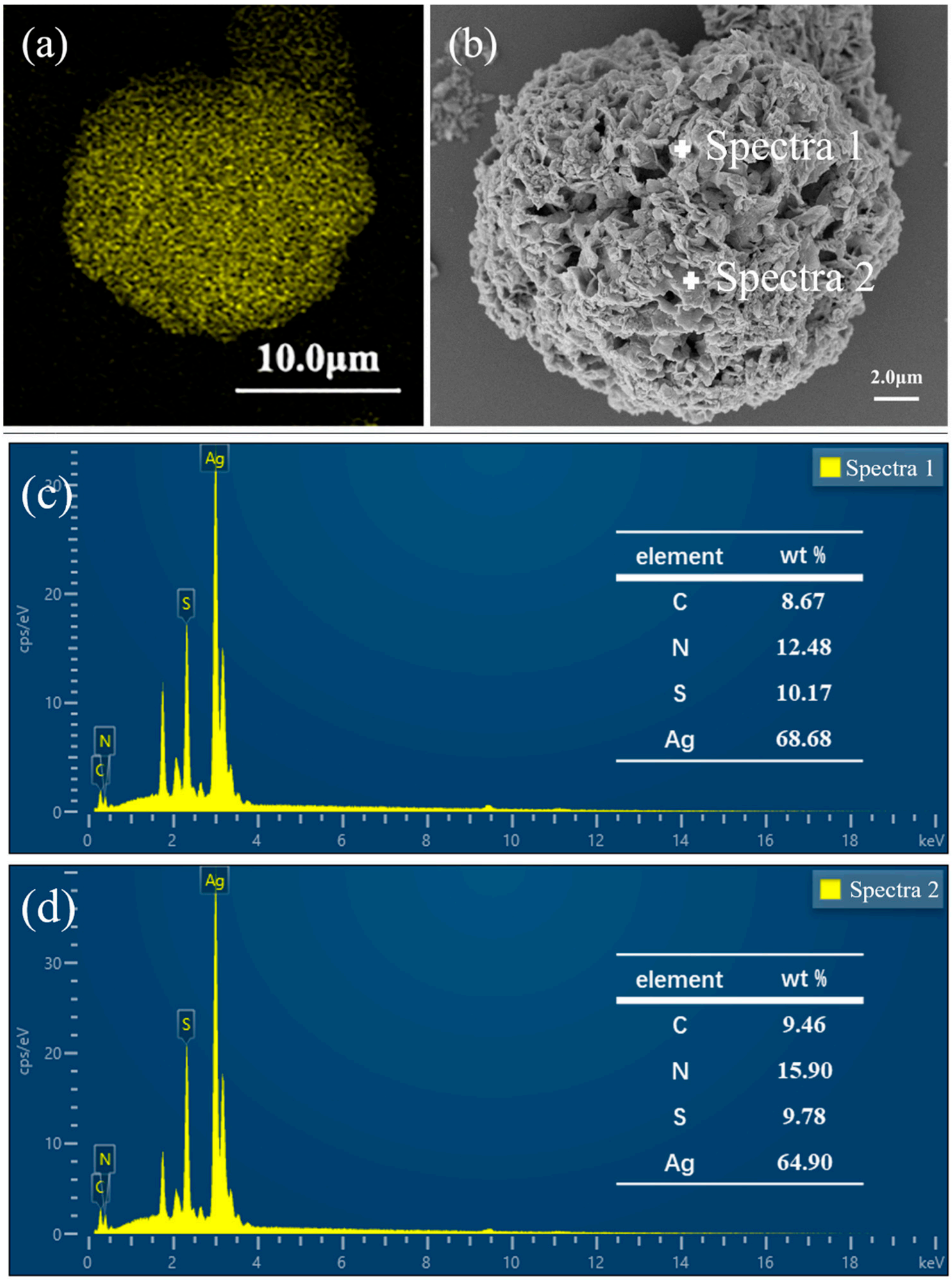

Figure S1. (a) The EDAX for silver ions distribution on FTFM surface. (b) two points on the FTFM surface for element mapping. (c,d) Elemental content of two points.

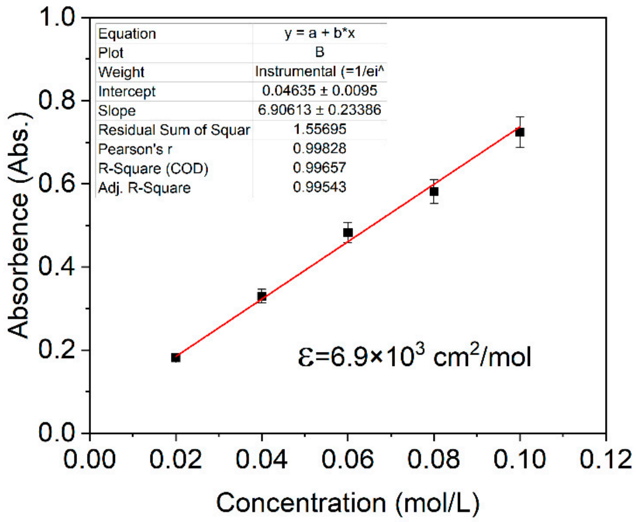

Figure S2. The calculation of the UV extinction coefficient of silver.

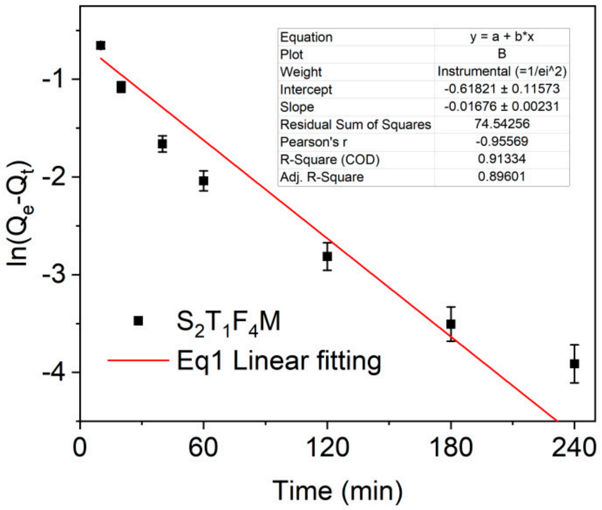

Figure S3. linear fitting between $ln(Q_e-Q_t)$ and adsorption time of $S_2T_1F_4M$ according to Equation (1) (main text).

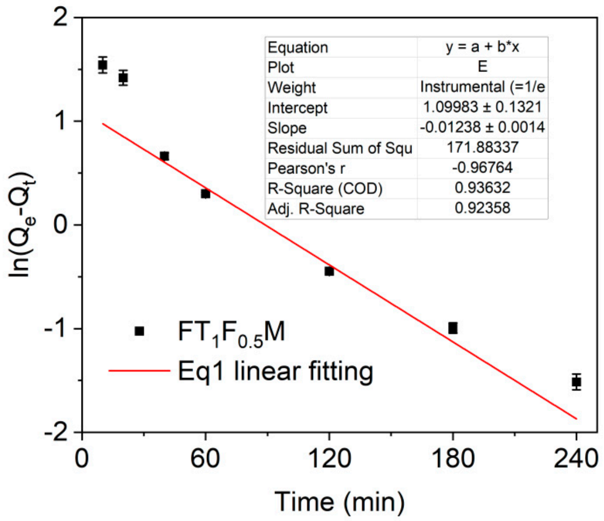

Figure S4. linear fitting between $ln(Q_e-Q_t)$ and adsorption time of $FT_1F_{0.5}M$ according to Equation (1) (main text).

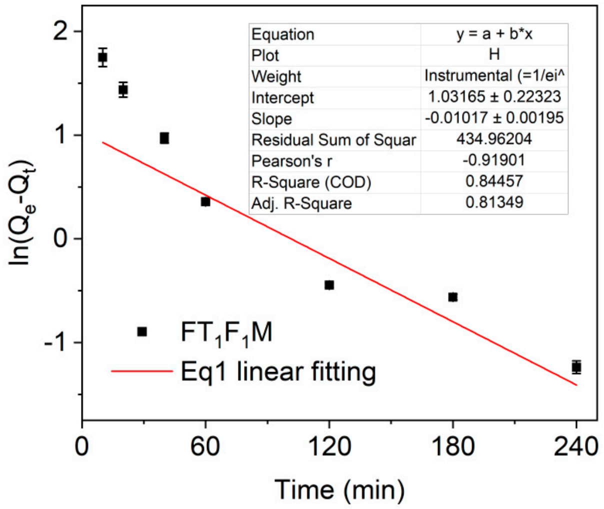

Figure S5. linear fitting between $ln(Q_e-Q_t)$ and adsorption time of FT_1F_1M according to Equation (1) (main text).

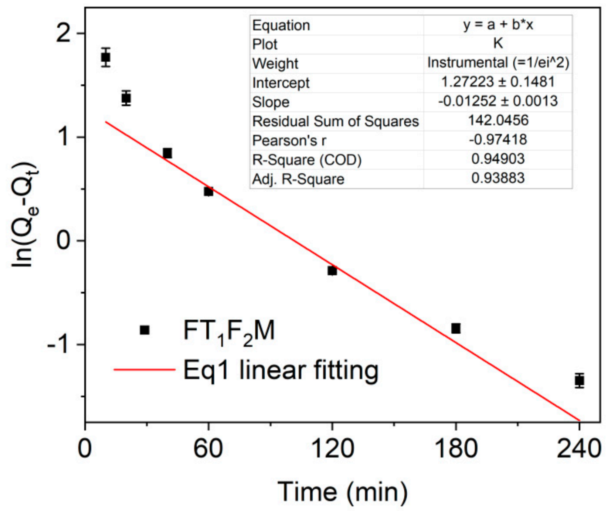

Figure S6. linear fitting between ln(Q_e-Q_t) and adsorption time of FT_1F_2M according to Equation (1) (main text).

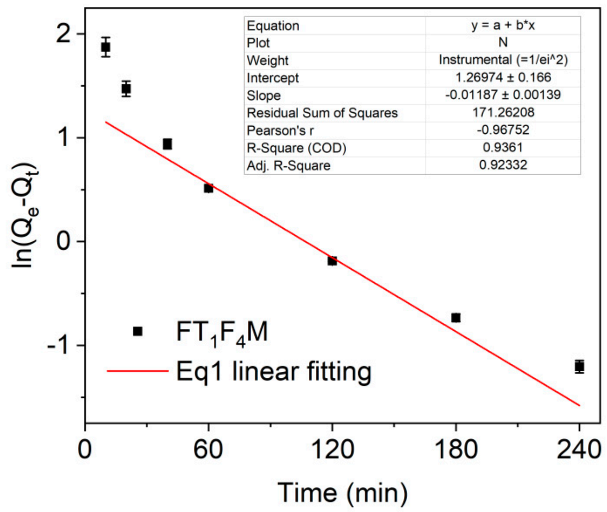

Figure S7. linear fitting between ln(Q_e-Q_t) and adsorption time of FT_1F_4M according to Equation (1) (main text).

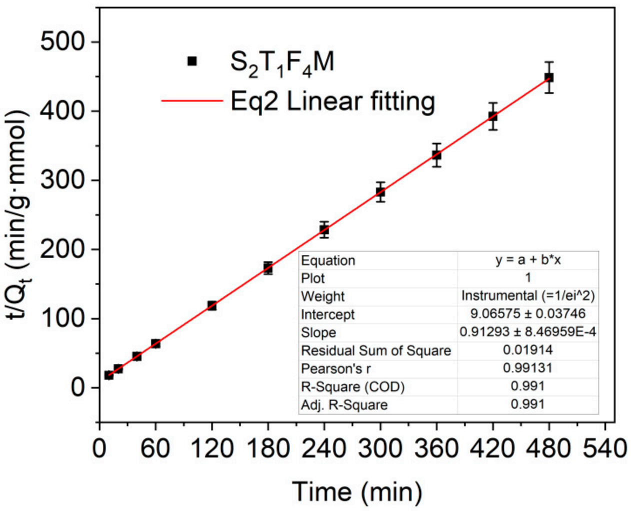

Figure S8. linear fitting between t/Q_t and adsorption time of $S_2T_1F_4M$ according to Equation (2) (main text).

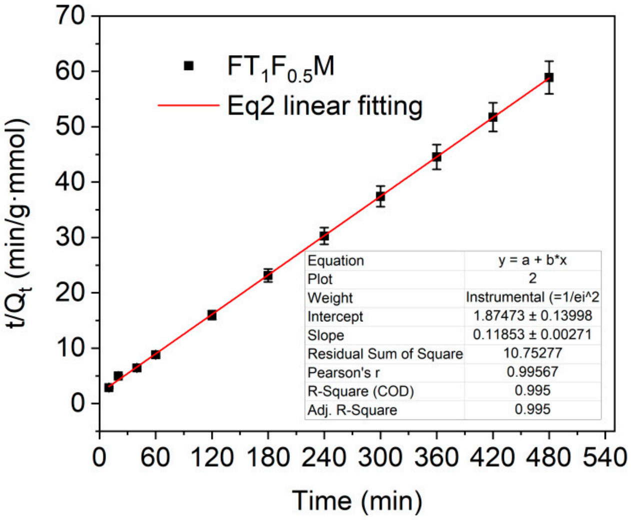

Figure S9. linear fitting between t/Q_t and adsorption time of $FT_1F_{0.5}M$ according to Equation (2) (main text).

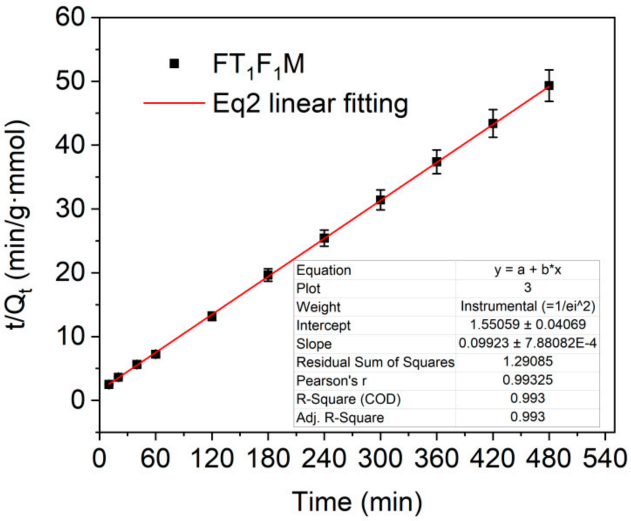

Figure S10. linear fitting between t/Q_t and adsorption time of FT_1F_1M according to Equation (2) (main text).

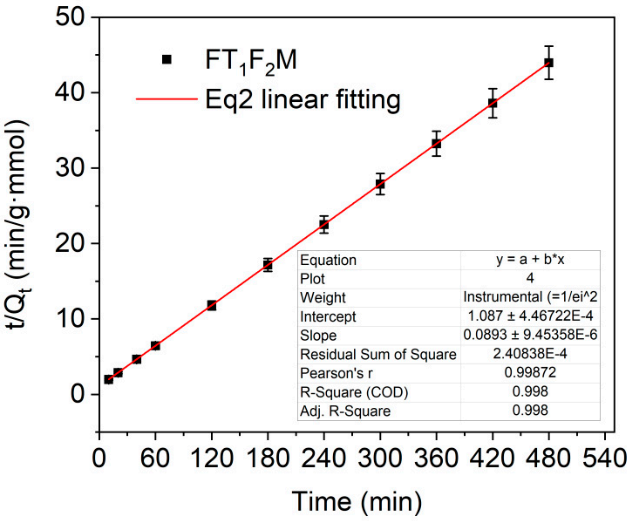

Figure S11. linear fitting between t/Q_t and adsorption time of FT_1F_2M according to Equation (2) (main text).

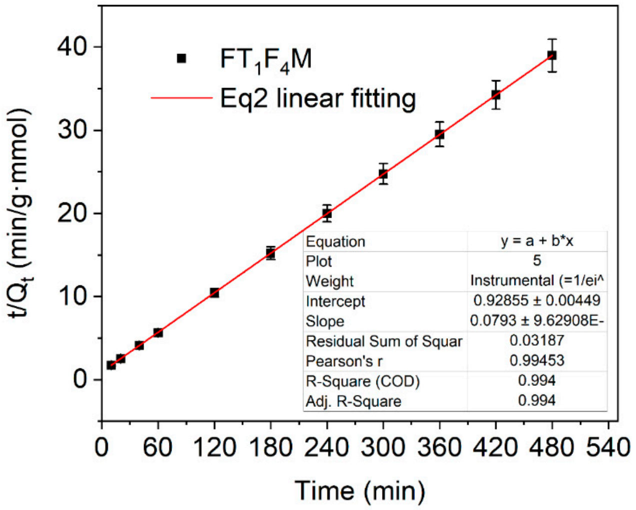

Figure S12. linear fitting between t/Q_t and adsorption time of FT_1F_4M according to Equation (2) (main text).

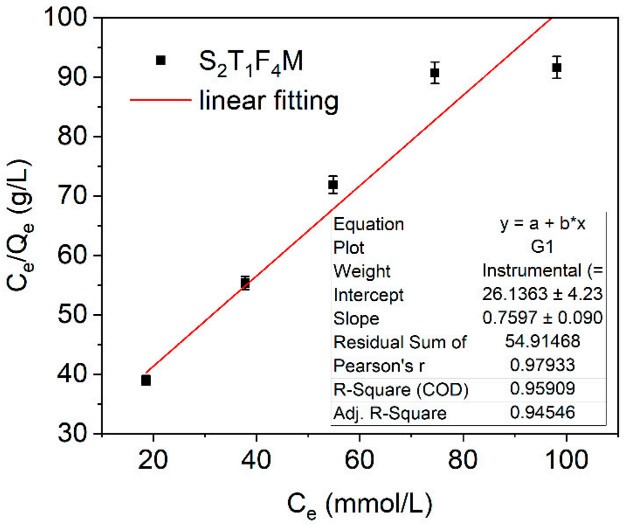

Figure S13. linear fitting between C_e/Q_e and Ce of $S_2T_1F_4M$ according to Equation (5) (main text).

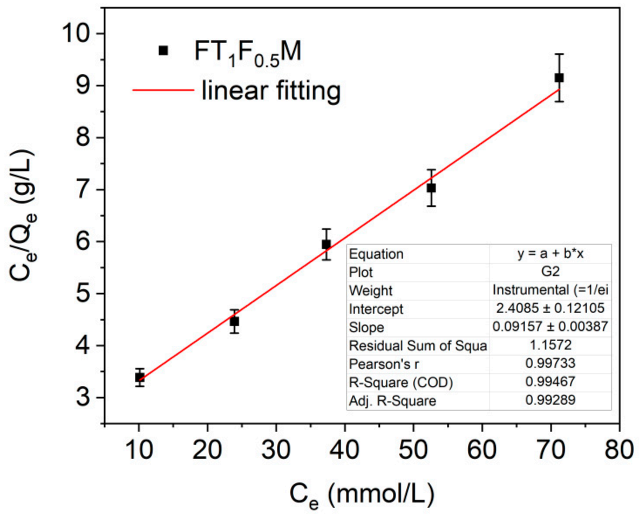

Figure S14. linear fitting between C_e/Q_e and Ce of $FT_1F_{0.5}M$ according to Equation (5) (main text).

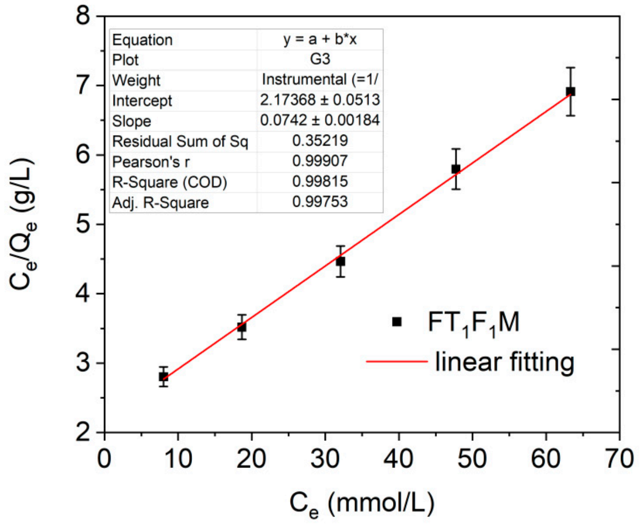

Figure S15. linear fitting between C_e/Q_e and Ce of FT_1F_1M according to Equation (5) (main text).

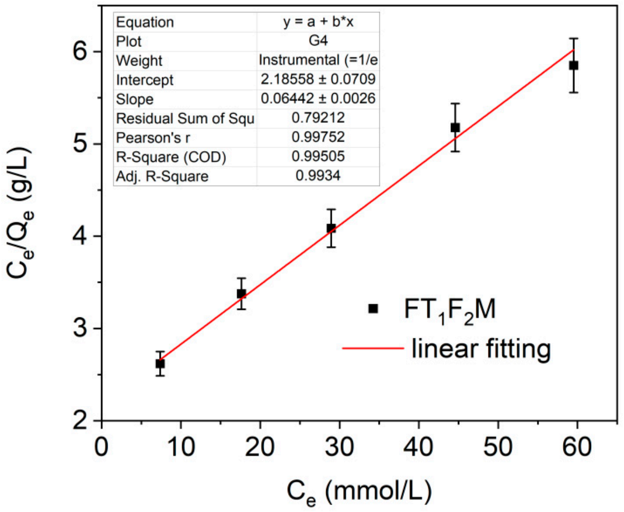

Figure S16. linear fitting between C_e/Q_e and Ce of FT_1F_2M according to Equation (5) (main text).

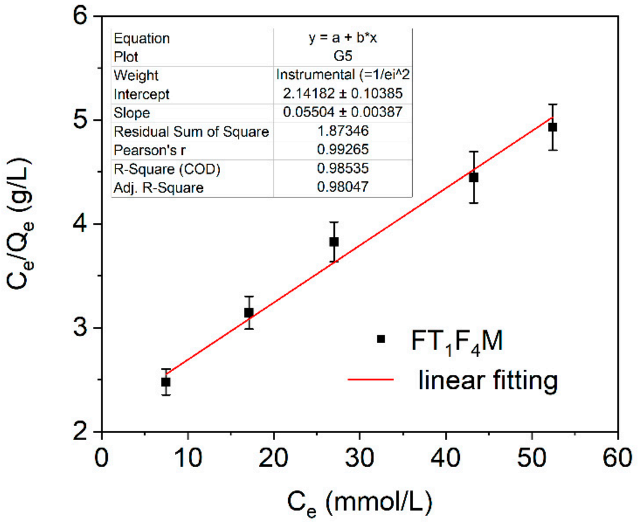

Figure S17. linear fitting between C_e/Q_e and Ce of FT_1F_4M according to Equation (5) (main text).

Table S1. Maximum adsorption capacities for adsorption of Ag+ onto various adsorbents.

Absorbent	Adsorption capacity (mmol/g)
Thiourea-modified chitosan resin	3.770[1]
zeolitic material from green tuff stone cake	1.100[2]
Halloysite nanotubes	1.018[3]
Sulfoethyl functionalized silica nanoparticle	0.203[4]
Flowerlike thiourea formaldehyde resin micro-spheres	18.17

References

1. Wang, L.; Xing, R.; Liu, S.; Yu, H.; Qin, Y.; Li, K.; Feng, J.; Li, R.; Li, P. Recovery of Silver (I) Using a Thiourea-Modified Chitosan Resin. *J. Hazard. Mater.* **2010**, *180*, 577–582. https://doi.org/10.1016/j.jhazmat.2010.04.072
2. Wajima, T. Synthesis of Zeolitic Material from Green Tuff Stone Cake and Its Adsorption Properties of Silver (I) from Aqueous Solution. *Microporous Mesoporous Mater.* **2016**, *233*, 154–162. https://doi.org/10.1016/j.micromeso.2016.01.020
3. Kiani, G. High Removal Capacity of Silver Ions from Aqueous Solution onto Halloysite Nanotubes. *Appl. Clay Sci.* **2014**, *90*, 159–164. https://doi.org/10.1016/j.clay.2014.01.010
4. Zhang, L.; Zhang, G.; Wang, S.; Peng, J.; Cui, W. Sulfoethyl Functionalized Silica Nanoparticle as an Adsorbent to Selectively Adsorb Silver Ions from Aqueous Solutions. *J. Taiwan Inst. Chem. Eng.* **2017**, *71*, 330–337. https://doi.org/10.1016/j.jtice.2017.01.001

Disclaimer/Publisher's Note: The statements, opinions and data contained in all publications are solely those of the individual author(s) and contributor(s) and not of MDPI and/or the editor(s). MDPI and/or the editor(s) disclaim responsibility for any injury to people or property resulting from any ideas, methods, instructions or products referred to in the content.
